# Clinical outcome of wild-type AmpC-producing Enterobacterales infection in critically ill patients treated with β-lactams: a prospective multicenter study

**DOI:** 10.1186/s13613-022-01079-5

**Published:** 2022-11-17

**Authors:** Roman Mounier, Ronan Le Guen, Paul-Louis Woerther, Mathieu Nacher, Clément Bonnefon, Nicolas Mongardon, Olivier Langeron, Eric Levesque, Séverine Couffin, Stéphanie Houcke, Michel Wolff, Ariane Roujansky, Caroline Schimpf, Armand Mekontso Dessap, Fabrice Cook, Keyvan Razazi, Hatem Kallel

**Affiliations:** 1 Département de Neuro-Anesthésie-Réanimation, GHU-Paris, Université de Paris, 1, Rue Cabanis, 75014 Paris, France; 2 Université de Paris, Paris, France; 3 INSERM U955, Équipe 15, Institut Mondor de la Recherche Biomédicale, Université Paris-Est-Créteil, Créteil, France; 4 Réanimation Polyvalente, Centre Hospitalier de Cayenne, Cayenne, Guyane Française France; 5 Département de Microbiologie, Hopitaux Universitaires Henri Mondor, Assitance Publique-Hôpitaux de Paris (AP-HP), Université Paris-Est-Créteil, Créteil, France; 6 Centre d’investigation Clinique, Antilles-Guyane (CIC INSERM 1424, Centre Hospitalier de Cayenne, Cayenne, Guyane Française France; 7 Service d’anesthésie-Réanimation Chirurgicale, DMU CARE, DHU A-TVB, Assistance Publique-Hôpitaux de Paris (AP-HP), Hôpitaux Universitaires Henri Mondor, 94010 Créteil, France; 8 Université Paris Est Créteil, Faculté de Santé, 94010 Créteil, France; 9 U955-IMRB, Equipe 03 “Pharmacologie et Technologies pour les Maladies Cardiovasculaires (PROTECT)”, Inserm, Univ Paris Est Créteil (UPEC), Ecole Nationale Vétérinaire d’Alfort (EnVA), 94700 Maisons-Alfort, France; 10 Département d’Anesthesie, Hospital Mignot, Versailles, France; 11 Service de Médecine Intensive-Réanimation, Assistance Publique-Hôpitaux de Paris (AP-HP), Hôpitaux Universitaires Henri-Mondor, 94010 Créteil, France; 12 Tropical Biome Et Immunopathologie CNRS UMR-9017, Inserm U 1, 019, Université de Guyane, Cayenne, Guyane Française France

**Keywords:** AmpC-producing Enterobacterales, AmpC β-lactamases, Third-generation cephalosporins, Infection, ICU

## Abstract

**Background:**

β-lactams are the main antibiotics used against wild-type AmpC-producing Enterobacterales (wtAE). However, they may fail or select AmpC-overproducing mutants. Our aim was to assess factors associated with clinical failure of β-lactams in the treatment of wtAE infection.

**Methods:**

From September 2017 to December 2020, we prospectively included all consecutive patients treated by definitive β-lactams therapy for wtAE infection in four university ICUs. Clinical failure was defined as inadequate response to antimicrobial therapy leading to death or to the switch for a broader-spectrum antibiotic.

**Results:**

177 patients were included and 29.4% progressed to clinical failure. *E. cloacae* was the most prevalent species (42.4%) and ventilator-associated pneumonia (VAP) was the most frequent wtAE infection (69.5%). Cefepime and cefotaxime were used as definitive antibiotic treatment in 42.9% and 27.7% of patients, respectively. Occurrence of AmpC-overproduction was documented in 5.6% of patients and was associated with clinical failure (*p* = 0.004). In multivariate analysis, VAP (*p* < 0.001, OR 11.58 [95% CI 3.11–43.02] and *K. aerogenes* (*p* = 0.030, OR 3.76 [95% CI 1.13–12.46]) were independently associated with clinical failure. Conversely, cefotaxime as definitive treatment was found inversely associated with the risk of clinical failure (*p* = 0.022, OR 0.25 [95% CI 0.08–0.82]). After inverse probability weighting, cefotaxime showed a 20% risk reduction of clinical failure (95% CI  5–35%, *p* = 0.007) whatever the location of infection, the SOFA score on the day of wtAE infection, or the bacterial species.

**Conclusions:**

Clinical failure in the treatment of wtAE infections is associated with the infection site and the causal microorganism. Additionally, cefotaxime use is probably protective against clinical failure in wtAE infection.

**Supplementary Information:**

The online version contains supplementary material available at 10.1186/s13613-022-01079-5.

## Introduction

Chromosomally-encoded AmpC-producing Enterobacterales (AE) include species with a natural resistance to aminopenicillins and first-generation cephalosporins (Supplementary introduction for information about what is an AmpC) [1]. Exposure to certain β-lactams can lead to in vitro and in vivo selection of high-level AmpC-producing mutants, resistant to third-generation cephalosporins (3GCs) [2]. The risk of AmpC overproduction seems to differ according to the AE species [3]. In addition, selection of resistance is often associated with poor outcome [4, 5].

Because of this risk, some authors recommend against 3GCs use, in case of infection due to wild-type AE (wtAE) [6, 7]. The main explanation is that 3GCs can select AmpC-overproducing AEs, making the responsible bacteria resistant to the ongoing antibiotic. In 38 ICU patients suffering from ventilator-associated pneumonia due to *E. cloacae* treated by cefotaxime, the clinical failure rate was 66% and the resistance selection was 47% [15]. Interestingly, the reported rates of emergence of 3GC resistance in patients treated with 3GCs were less than or equal to 10% [4, 8–11].

However, all these studies contain methodological bias [12], and confusion persists between the clinical outcome and the emergence of resistance. Consequently, conclusions from these studies drove to an overuse of large spectrum β-lactams in wtAE infections [2] with high risk of emergence of resistances to other antibiotics [13]. In addition, only few studies have focused on ICU patients and on the clinical cure of infection.

The primary endpoint of our study was to assess the prevalence of clinical failure in ICU patients with wtAE infections treated by β-lactams as definitive therapy. The secondary endpoints were to identify risk factors for clinical failure and those for the emergence of AmpC overproduction in the same context.

## Methods

We conducted a prospective multicenter, observational study in four university medical and surgical ICUs in France. We included all consecutive patients hospitalized between September 2017 and December 2020 with documented wtAE infection and treated, as definitive therapy, with a β-lactam to which the strain was susceptible. Moreover, empirical antibiotic therapy, whatever the molecule used, should be active in vitro on the wtAE, and the definitive therapy should be active on all pathogens isolated in the clinical specimen. Exclusion criterion was death from any cause, within 48 h of the start of the antimicrobial therapy.

This study was approved by the Institutional Ethical Committee CEADM Claude Galien (no. 2016–046).

### Definitions

Infections were defined according to the International Sepsis Forum consensus conference [14]. Probable or possible pneumonia was defined by a new and persistent infiltrate on chest radiography associated with at least one of the following [15]: (1) fever (central temperature ≥ 38.3 °C) or hypothermia (< 36.0 °C); (2) leukocytosis (> 10,000 WBC/mm^3^) or leukopenia (≤ 4000 WBC/mm^3^); (3) increase in volume or new onset of purulent sputum; for patients experiencing acute respiratory distress syndrome or other pre-existing/persisting pulmonary infiltrates for whom it was difficult to demonstrate deterioration of the radiologic images, at least one of the three preceding criteria sufficed for inclusion; and (4) positive quantitative cultures of pulmonary secretion samples, obtained by a protected telescopic catheter in intubated patients (significant threshold ≥ 10^3^ cfu/mL). Ventilator-associated pneumonia (VAP) was defined as pneumonia occurring in patients under mechanical ventilation (MV) for more than 48 h [16].

wtAE infection refers to an infection for which the microbiological sample grows to wtAE above the retained threshold, whether the culture was mono- or poly-microbial. Appropriate definitive β-lactam therapy was defined as the use of a β-lactam to which the strain was susceptible. Susceptibility was defined according to the European Committee on Antimicrobial Susceptibility Testing (EUCAST) recommendations [17].

AE species included *Enterobacter cloacae, Klebsiella aerogenes, Serratia marcescens, Citrobacter freundii, Providencia* spp.*, Hafnia alvei, and Morganella morganii.* AE were considered “wild type” if they displayed a low-level expression of AmpC enzymes, with retained susceptibility to 3GCs using the EUCAST breakpoint of ≤ 1 μg/mL [17].

Clinical cure was defined as complete or partial resolution of signs and symptoms of infection, so that no further antimicrobial therapy was necessary during the 5 days following treatment discontinuation [18, 19]. Clinical failure was defined as persistence of signs and symptoms of infection leading to the switch for a broader-spectrum antibiotic, a new clinical sample, or death, from 48 h after treatment introduction until 5 days after treatment discontinuation. The clinical response was assessed by the medical team. The classification used to define the β-lactam spectrum, mainly the broader spectrum, is that proposed by Weiss et al. [20].

Recurrent infection was defined as a new infection with the same strain regardless of the phenotype, at the same site, more than 5 days after antimicrobial therapy discontinuation. A new infection was defined as an infection with the same strain regardless of the phenotype, at a different site, more than 5 days after antimicrobial therapy discontinuation.

Microbiological failure was defined as the isolation of the same wtAE growing above the threshold in the sample culture obtained at the end of the antimicrobial treatment, or the culture growing with the same AE overproducing AmpC, regardless of the threshold. Mortality refers to all-cause mortality. Definitive therapy was the treatment administered for ≥ 50% of the total treatment course [9].

Patients were categorized as immunocompromised if they received a previous solid-organ transplant or bone marrow transplant, chemotherapy within the past 6 months, were infected with the human immunodeficiency virus, had a documented congenital immunodeficiency, received any immunomodulatory agent within the past 30 days, or received at least 10 mg of corticosteroids for > 14 days.

### Study protocol

Clinical samples were sent to microbiology for analysis, where one microbiologist centralized all positive AE results. The microbiologist contacted the investigators each week to communicate the list of patients with wtAE-positive specimens.

The management of patients was left to the discretion of the clinician in charge: the decision of the initial sampling, the choice of the antimicrobial therapy, the duration of treatment, and the decision to modify the antimicrobial therapy or to perform a new microbiological sampling. In case of poor clinical evolution, the intensive care physicians met with an infectious disease specialist to reassess the treatment.

### Laboratory methods

Clinical samples were processed and cultured according to the French Microbiology Society recommendations [21]. Antibiotic susceptibility testing was performed by MicroScan WalkAway [22] (Beckman-Coulter®) for urine samples and by disk diffusion technique for other sample types, according to CASFM-EUCAST guidelines (see Additional file 1: methods) [23]. The susceptibility results to the tested antibiotics were obtained by comparing the minimal inhibitory concentration (MIC) or the measured inhibition zone diameters to the CASFM–EUCAST clinical breakpoints [23]. Strains categorized as sensitive to cefotaxime and ceftazidime were considered to have a basal level of AmpC. In case of resistance to 3GCs, the strain was tested on Mueller Hinton agar plates loaded with 250 mg/l cloxacillin [24] (Biorad®), an AmpC inhibitor, in order to determine whether the displayed phenotype was the consequence of an AmpC hyperproduction and/or the production of an Extended Spectrum β-Lactamase (ESBL). Infections caused by microorganisms with AmpC overproduction or ESBL production, and other non-wtAE, were not included in this study.

### Statistical analysis

Statistical analyses were performed using Excel (2007), IBM SPSS Statistics for Windows, version 24 (IBM Corp., Armonk, NY, USA), and STATA 16 (STATA Corp, College Station, Texas). Results were reported as the number of patients for whom the data were recorded (Nb), the median and interquartile range [IQR], or numbers with percentages. To compare qualitative variables, we used the Fisher exact test. Continuous variables were compared using the Mann–Whitney U test. Variables associated with clinical failure at the 0.2 level by univariate analysis were entered into the stepwise logistic regression model. We calculated the odd ratio (OR) and the 95% confidence interval [95% CI]. Treatment effects estimation used inverse probability weighting to control for potential systematic differences in the allocation of treatment between patients. All statistical tests were two tailed, and *p* ≤ 0.05 was considered significant.

## Results

From September 2017 to December 2020, the microbiological laboratory reported 355 clinical samples positive to wtAE in 282 ICU patients. There was no sporadic outbreak due to one of these bacteria during the study period. Twenty-three patients were excluded because they died within 48 h of antibiotic treatment, 8 because of antimicrobial therapy without β-lactam, 6 for missing data, and 68 patients who did not receive antibiotics. Overall, 177 patients were included (Additional file 1: Fig. S1). The incidence of clinical failure was 29.4% (52/177).

### Baseline characteristics

Baseline demographic and clinical characteristics at ICU admission were similar whatever the clinical outcome (Table 1). Severity at admission measured by SAPSII and SOFA scores was similar between groups.

**Table 1:** Baseline characteristics of patients at ICU admission

	All population (*n* = 177)		Clinical failure (*n* = 52)		Clinical cure (*n* = 125)		*p*
	Nb	Results	Nb	Results	Nb	Results	
Gender (male)	177	134 (75.7%)	52	42 (80.8%)	125	92 (73.6%)	0.311
Age (years)	177	64 (50–70)	52	65 (50–70)	125	64 (50–71)	0.928
Past medical history							
Hospitalization during the last 12 months	175	72 (41.1%)	52	21 (40.4%)	123	51 (41.5%)	0.895
Hospitalization delay (months)	74	1 (1–2)	21	1 (1–3)	53	1 (1–1)	**0.03**
ATBs during the last 3 months	166	31 (18.7%)	49	9 (18.4%)	117	22 (18.8%)	0.948
Immunosuppression	177	61 (34.5%)	52	14 (26.9%)	125	47 (37.6%)	0.173
Corticosteroids	61	50 (82%)	14	12 (85.7%)	47	38 (80.9%)	0.678
Immunosuppressive therapy	61	10 (16.4%)	14	2 (14.3%)	47	8 (17%)	0.808
Hemopathy	61	1 (1.6%)	14	0 (0%)	47	1 (2.1%)	0.582
Extravascular device	177	7 (4%)	52	2 (3.8%)	125	5 (4%)	0.962
Endovascular device	177	40 (22.6%)	52	9 (17.3%)	125	31 (24.8%)	0.278
Diabetes mellitus	177	46 (26%)	52	17 (32.7%)	125	29 (23.2%)	0.19
McCabe score	176	1 (0–2)	51	1 (0–2)	125	1 (0–2)	0.933
Delay from hospitalization to ICU (days)	174	0 (0–1)	50	0 (0–1)	124	0 (0–0)	0.408
ICU							
Surgery at admission	176	57 (32.4%)	52	16 (30.8%)	124	41 (33.1%)	0.767
Infection at admission	175	69 (39.4%)	51	17 (33.3%)	124	52 (41.9%)	0.29
SAPS II score	140	37 (27–49)	42	37 (27–54)	98	37.5 (27–49)	0.747
SOFA score at admission	131	6 (3–8)	41	5 (3–8)	90	7 (3–8)	0.972

Hospitalization delay is the delay between the last hospitalization and this hospitalization. Intravascular devices include all implanted medical devices with an intravascular portion. Extravascular devices include all medical devices implanted without contact with blood

ATBs: antibiotics; ICU: Intensive Care Unit; Nb: number of available values for the data; SAPS II: Simplified Acute Physiology Score II; SOFA: Sequential Organ Failure Assessment

There was no difference between the groups in terms of prior ICU infections (37.3%), nor in terms of the prior antibiotics exposure (see Additional file 1: Table S1).

The patients’ management during the ICU stay and before the occurrence of the wtAE infection is shown in Table 2.

**Table 2:** Management of patients during ICU course

	All population (*n* = 177)		Clinical failure (*n* = 52)		Clinical cure (*n* = 125)		*p*
	Nb	Results	Nb	Results	Nb	Results	
ICU management							
MV	177	151 (85.3%)	52	49 (94.2%)	125	102 (81.6%)	**0.031**
Duration of MV (days)	149	18 (9–31)	48	19 (12–31)	101	18 (8–30)	0.109
Renal replacement therapy	177	49 (27.7%)	52	23 (44.2%)	125	26 (20.8%)	**0.002**
Chest drainage	177	20 (11.3%)	52	8 (15.4%)	125	12 (9.6%)	0.268
ECMO	177	28 (15.8%)	52	13 (25%)	125	15 (12%)	**0.031**
EVD	177	13 (7.3%)	52	3 (5.8%)	125	10 (8%)	0.604
Surgical procedure during ICU course	177	82 (46.3%)	52	23 (44.2%)	125	59 (47.2%)	0.718

ECMO: Extracorporeal Membrane Oxygenation; EVD: External ventricular Drain; ICU: Intensive Care Unit; MV: mechanical ventilation (invasive); Nb: number of available values for the data

### Primary endpoint

**Fig. 1:**
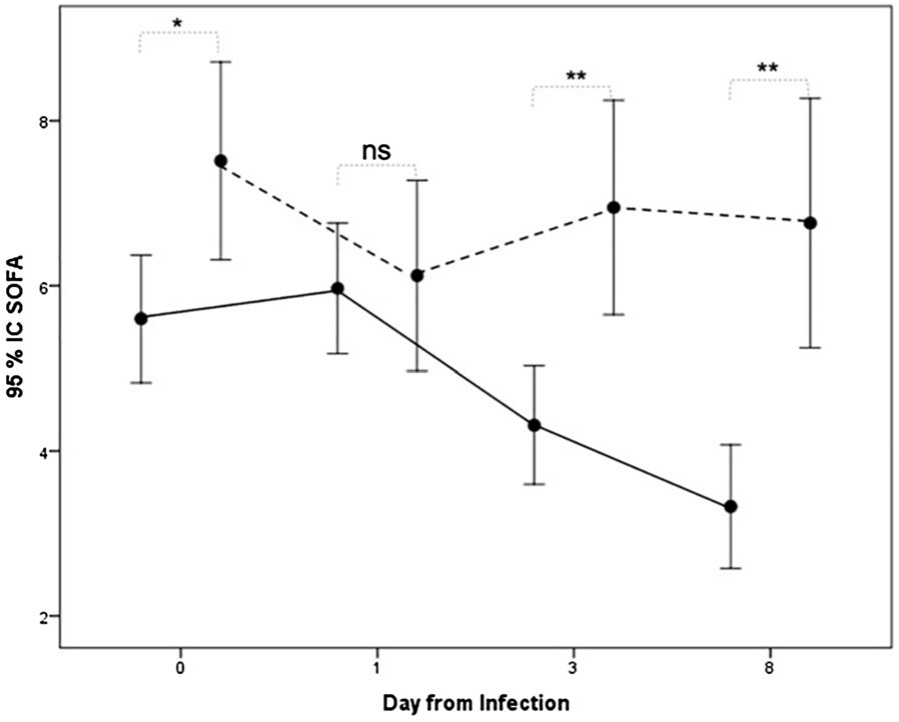
Evolution of SOFA score between the two groups, failure and clinical success, from wtAE infection and as a function of time. The dotted line represents the clinical failure group and the solid line represents the clinical success group. *p < 0.02, ** ≤ 0.001

Patients from the clinical failure group were more seriously ill, as expressed by a higher SOFA score on the day of infection (8 vs. 6, *p* = 0.028) (Table 3). This difference in the SOFA score increased between the two groups over time (Fig. 1).

**Table 3:** Characteristics and outcome at inclusion (Day 1 of wild-type AmpC-producing Enterobacterales infection)

	All population (*n* = 177)		clinical failure (*n* = 52)		clinical cure (*n* = 125)		*p*
	Nb	Results	Nb	Results	Nb	Results	
Number of ICU infection before inclusion	177	0 [0–1]	52	0 [0–1]	125	0 [0–1]	0.283
Time between previous ICU infection and inclusion (days)	66	8 [4–14]	20	8 [6–13]	46	8 [4–16]	0.742
Time between ICU admission and inclusion (days)	176	7 [2–11]	51	7 [4–11]	125	6 [2–11]	0.155
SOFA score at day 0 of infection	133	6 [3–9]	41	8 [5–10]	92	6 [3–8]	**0.014**
MV during infection	177	144 (81.4%)	52	49 (94.2%)	125	95 (76%)	**0.005**
Renal replacement therapy during infection	177	38 (21.5%)	52	20 (38.5%)	125	18 (14.4%)	**0.000**
Urinary catheter during infection	177	171 (96.6%)	52	52 (100%)	125	119 (95.2%)	0.108
Catheter during infection	177	132 (74.6%)	52	46 (88.5%)	125	86 (68.8%)	**0.006**
Number of catheters	177	2 [0–2]	52	2 [2–2]	125	2 [0–2]	**0.000**
Catheters at the end of AMB therapy	177	89 (50.3%)	52	35 (67.3%)	125	54 (43.2%)	**0.003**
Number of catheters at the end of AMB therapy	177	1 [0–2]	52	2 [0–2]	125	0 [0–2]	**0.001**
Site of wtAE infection							
Lung	177	132 (74.6%)	52	48 (92.3%)	125	84 (67.2%)	**0.000**
Ventilator-associated pneumonia	132	123 (93.2%)	52	47 (90.4%)	125	76 (60.8%)	**0.000**
Skin and Soft tissue	177	14 (7.9%)	52	2 (3.8%)	125	12 (9.6%)	0.196
Abdomen	177	11 (6.2%)	52	1 (1.9%)	125	10 (8%)	0.127
Primary bacteremia	177	5 (2.8%)	52	0 (0%)	125	5 (4%)	0.143
CSF	177	5 (2.8%)	52	0 (0%)	125	5 (4%)	0.323
Others	177	10 (5.6%)	52	1 (1.9%)	125	9 (7.2%)	0.285
Microbiological samples after completion of AMB therapy	177	85 (48%)	52	33 (63.5%)	125	52 (41.6%)	**0.008**
Microbiological failure	85	27 (31.8%)	33	22 (66.7%)	52	5 (9.6%)	**0.000**
Overproduced cephalosporinases	26	5 (19.2%)	33	5 (15.2%)	52	0 (0%)	**0.007**
Recurrent AE infection	177	24 (13.6%)	52	8 (15.4%)	125	16 (12.8%)	**0.647**
Time to recurrent infection (days)	28	11.5 [5–18]	5	12 [6–21]	15	16 [12–23]	0.358
Recurrent infection with overproduced cephalosporinases	26	3 (15.8%)	5	2 (40%)	13	1 (7.7%)	0.099
New AE infection	177	8 (4.5%)	52	2 (3.8%)	125	6 (4.8%)	**0.781**
Time between the first and the second AE infection (days)	8	30 [24–48]	2	48 [35–60]	6	30 [26–43]	0.737
Overproduced cephalosporinases	7	2 (28.6%)	1	0 (0%)	6	2 (33.3%)	0.495
Outcome							
AmpC+	177	10 (5.6%)	52	7 (13.5%)	125	3 (2.4%)	**0.004**
Death during ICU stay	177	68 (38.4%)	52	36 (69.2%)	125	32 (25.6%)	**0.000**
Length of ICU stay (days)	170	23 [14–39]	52	24 [15–32]	118	22 [14–39]	0.625
Death during hospitalization	177	73 (41.2%)	52	38 (73.1%)	125	35 (28%)	**0.000**
Time between hospitalization and death (days)	72	28 [16–45]	36	21 [13–35]	36	34 [22–48]	**0.006**
Time between wtAE infection and discharge from ICU (days)	168	17 [8–26]	51	14 [8–26]	117	17 [9–28]	0.411
Death within the first five days of AMB therapy	176	7 (4%)	51	7 (13.7%)	125	0 (0%)	**0.000**
Withdrawal or withholding of life support	68	40 (58.8%)	36	16 (44.4%)	32	24 (75%)	**0.011**

Catheters include arterial and central venous catheters. Effective antimicrobial therapy includes empirical and definitive antimicrobial therapy. AmpC+ corresponds to all overproducing-AmpC AE occurring during the treatment of the infection until discharge from the ICU. Mortality refers to all-cause mortality

wtAE: wild-type AmpC-producing Enterobacterales; ICU: Intensive Care Unit; Nb: number of available values for the data; MV: mechanical ventilation (invasive); SOFA: Sequential Organ Failure Assessment

Pneumonia and VAP were associated with clinical failure (48/52 vs. 84/125 and 47/52 vs. 76/125, respectively, both *p* < 0.001). wtAE infections were mainly pneumonia (132/177, 74.6%) which were VAP in the majority of cases (123/132, 93.2%) (Table 3).

Regarding species, *K. aerogenes* was associated with clinical failure (17/52, 32.7% vs. 15/125, 12%; *p* = 0.001) (Table 4).

**Table 4:** Microorganisms responsible for infections and antimicrobial therapy

	All population (*n* = 177)		Clinical failure (*n* = 52)		Clinical success (*n* = 125)		*p*
	Nb	Results	Nb	Results	Nb	Results	
Monomicrobial infection	177	71 (40.1%)	52	24 (46.2%)	125	47 (37.6%)	0.29
*E. cloacae*	177	75 (42.4%)	52	18 (34.6%)	125	57 (45.6%)	0.178
*K. aerogenes*	177	32 (18.1%)	52	17 (32.7%)	125	15 (12%)	**0.001**
*S. marcescens*	177	43 (24.3%)	52	13 (25%)	125	30 (24%)	0.888
*C. freundii*	177	11 (6.2%)	52	2 (3.8%)	125	9 (7.2%)	0.4
*M. morganii*	177	16 (9%)	52	1 (1.9%)	125	15 (12%)	**0.003**
*H. alvei*	177	14 (7.9%)	52	5 (9.6%)	125	9 (7.2%)	0.588
*P. aeruginosa*	177	19 (10.7%)	52	4 (7.7%)	125	15 (12%)	0.399
*S. aureus*	177	23 (13%)	52	4 (7.7%)	125	19 (15.2%)	0.176
Bacterial inoculum (cfu/mL)	113	10 [1–1, 000]	45	10^4^ [10^3^–775 × 10^3^]	91	5 × 10^4^ [10^3^–10^6^]	0.261
Empirical therapy	177	177 (100%)	52	52 (100%)	125	125 (100%)	
Combination therapy	177	166 (93.8%)	47	12 (25.5%)	119	30 (25.2%)	0.966
Strains susceptible to the empirical AMB therapy	177	166 (93.8%)	52	47 (90.4%)	125	119 (95.2%)	0.227
wtAE susceptible to the β-lactam included in empirical therapy	177	163 (92.1%)	52	45 (86.5%)	125	118 (94.4%)	0.078
Duration of empirical antimicrobial therapy (days)	103	2 [2, 3]	33	2 [1–3]	70	2 [2, 3]	0.188
Cefotaxime	177	31 (17.5%)	52	5 (9.6%)	125	26 (20.8%)	0.075
Piperacillin–tazobactam	177	55 (31.1%)	52	14 (26.9%)	125	41 (32.8%)	0.442
Cefepime	177	61 (34.5%)	52	19 (36.5%)	125	42 (33.6%)	0.708
Imipenem	177	8 (4.5%)	52	3 (5.8%)	125	5 (4%)	0.606
Meropenem	177	6 (3.4%)	52	3 (5.8%)	125	3 (2.4%)	0.259
Carbapenem	177	14 (7.9%)	52	6 (11.5%)	125	8 (6.4%)	0.249
Amikacin	177	42 (23.7%)	52	12 (23.1%)	125	30 (24%)	0.895
Definitive antimicrobial therapy							
Duration of antimicrobial therapy (days)	169	7 [6–10]	51	7 [6–9]	118	7 [7–11]	0.11
Piperacillin	177	18 (10.2%)	52	5 (9.6%)	125	13 (10.4%)	0.875
Cefotaxime	177	49 (27.7%)	52	8 (15.4%)	125	41 (32.8%)	**0.018**
Piperacillin–tazobactam	177	21 (11.9%)	52	6 (11.5%)	125	15 (12%)	0.931
Cefepime	177	76 (42.9%)	52	27 (51.9%)	125	49 (39.2%)	0.119
Imipenem	177	3 (1.7%)	52	2 (3.8%)	125	1 (0.8%)	0.153
Meropenem	177	10 (5.6%)	52	4 (7.7%)	125	6 (4.8%)	0.448
Carbapenem	177	13 (7.3%)	52	6 (11.5%)	125	7 (5.6%)	0.168

Bacterial inoculum concerns only samples collected by telescopic catheters protected in ventilator-associated pneumonia. Combination therapy means the combination of two antibiotics active in vivo, on the wild-type AmpC-producing Enterobacterales. Regarding empirical therapy, the susceptibility of strains to empirical antimicrobial therapy concerns all strains found in the sample, whether they are wtAE or not

AMB: antimicrobial; wtAE: wild-type AmpC-producing Enterobacterales; Nb: number of available values for the data

Regarding the definitive β-lactam therapy, only 3GCs were associated with clinical cure (8/52, 15.4% in “clinical failure” vs. 41/125, 32.8% in “clinical cure” groups, *p* = 0.018). All but one 3GCs were cefotaxime.

All-cause mortality rate was 38.4%. ICU and hospital death were higher in the “clinical failure” group (*p* < 0.001).

### Multivariate analysis

Six relevant variables were included in the multivariate analysis: the SOFA score on the day of AE infection, *E. cloacae*, *K. aerogenes*, VAP, and cefotaxime and cefepime used as a definitive treatment. Of these, VAPs (*p* < 0.001, OR 11.58 [95% CI 3.11–43.02]) and *K. aerogenes* (*p* = 0.030, OR 3.76 [95% CI 1.13–12.46]), were independently associated with clinical failure. Cefotaxime as a definitive treatment was inversely associated with “clinical failure” (*p* = 0.022, OR 0.25 [95% CI 0.08–0.82]). After inverse probability weighting to remove confounding by SOFA score, VAP, *E. cloacae*, and *K. aerogenes* the average treatment effect in the population showed that those who received cefotaxime had a 20% risk reduction of clinical failure (95% CI 5–35%) relative to those who did not receive cefotaxime (*p* = 0.007).

### Secondary endpoint

#### Empirical antibiotics

Empirical antibiotics were similar in both groups, without difference on outcome (Table 4). Empirical β-lactams were effective against wtAE in 92.1% (163/177) of cases, without difference between groups.

#### Microbiological failure

Microbiological failure was present in 66.7% (22/33) of cases in the clinical failure group vs. 9.6% (5/52) in the “clinical cure” group, *p* < 0.001 (Table 3). Overproduced AmpC was found in only 5 cases, all of them in the “clinical failure” group.

#### Recurrent and new AE infection

Recurrent (24/177; 13.6%) and new AE infections (8/177; 4.5%) were recorded in both groups without difference (Table 3). Recurrent (5/26, 19.2%) or new AE infections (2/7, 28.6%) with overproduced AmpC were similar in both groups.

#### AmpC overproduction

For this analysis, the population was divided into two groups, those who selected an AmpC-overproducing strain (in any microbiological sample between inclusion and discharge from the ICU, *n* = 10), and those without selection (*n* = 167) (Additional file 1: Table S2). Baseline characteristics were similar, including the number of previous infections and the different classes of antibiotics received. AmpC overproduction was associated with clinical and microbiological failure (7/52 (13.5%) vs. 3/125 (2.4%), *p* = 0.004 and 6/8 (75%) vs. 21/77 (27%), *p* = 0.006, respectively), and with a longer ICU stay (45 [31–59] vs. 22 days [13–35], *p* = 0.004). All overproducing-AmpC AE were isolated from VAPs (10/10). Regarding the antibiotics used as definitive therapy, combination therapy and cefepime were associated with a lower risk of AmpC overproduction (*p* = 0.042 and *p* = 0.03, respectively).

Among the overproducing-AmpC strains, two *K. aerogenes*, two *E. cloacae,* and one *S. marcescens* were responsible for clinical failure. One *K. aerogenes*, one *E. cloacae,* and one *S. marcescens* were responsible for recurrent infection. Two *E. cloacae* caused new infection. Finally, overproduced cephalosporinase were selected for 9% (3/32) of *K. aerogenes*, 7% (5/75) of *E. cloacae* and 5% (2/43) of *S. marcescens*.

## Discussion

One third of critically ill patients with wild-type AmpC-producing Enterobacterales infection and treated with definitive appropriate β-lactam antimicrobial therapy experienced clinical failure. Clinical failure was associated with VAPs and *K. aerogenes*. Moreover, clinical failure was associated with ICU and hospital death, microbiological failure, and selection of AmpC-overproducing AE during treatment. Surprisingly, cefotaxime was inversely associated with the risk of clinical failure. All AmpC-overproducing AE were isolated from respiratory tract samples in patients with VAP.

Most studies focused on the emergence of derepressed AmpC, and on 28-day mortality [8, 25]. However, only few studies reported the clinical failure rates in AE infections in ICU patients [26, 27]. Füssle et al*.*, studying wtAE-VAPs treated by cefotaxime, reported a clinical failure rate of 66% [26]. Arthur et al*.* reported clinical failure in the treatment of VAP higher than 50% [27]. In our study, we found 29.4% of clinical failure in ICU patients. VAPs accounted for 69.5% of infections and were strongly associated with clinical failure (*p* < 0.001, OR 11.58 [95% CI 3.11– 43.02]). Lung infection is characterized by a high inoculum that cannot be surgically reduced [10]. Of note, all the AmpC-overproducing AE found in our study were initially isolated from the lungs. Overall, among reported AE infections, pneumonia is the most prevalent (up to 40%) [8, 25, 28, 29] and is commonly associated with mortality [8, 25].

Unexpectedly, we found that the use of cefotaxime as definitive therapy was associated with a lower clinical failure rate. Interestingly, most studies found no difference in outcome according to the β-lactam used in the treatment of wtAE infections [5, 9, 25, 31–33]. As in our study, Siedner et al*.* found that among 368 patients with *Enterobacter* spp. bacteremia, the lowest rate of mortality was observed in those treated with ceftriaxone [34]. Our result is surprising although verified by various statistical analyses, multivariate analysis, and matching on different variables, such as severity on the day of infection (inverse probability weighting). It can be influenced by the observational nature of our study and by differences between groups. For example, patients treated with cefotaxime were ventilated for a lesser time, which may reflect a lesser severity. Another explanation could be due to the cefotaxime itself. IDSA experts suggests that when wild-type *E. cloacae, K. aerogenes, or C. freundii* are recovered in clinical cultures, ceftriaxone or ceftazidime treatment should be avoided, because these strains are likely to overexpress AmpC [32]. This suggestion is based on studies using ceftriaxone with only few data on cefotaxime in this context. Indeed, ceftriaxone has been widely associated with the selection of strains overexpressing AmpC [32, 35]. Moreover, these studies present many weaknesses, such as not reporting the mechanism associated with ceftriaxone non-susceptibility (e.g., ESBL production) or using pre-2010 CLSI ceftriaxone breakpoints (i.e., ceftriaxone MICs ≤ 8 μg/mL), making translation of the prevalence estimates to current CLSI ceftriaxone susceptibility breakpoint (≤ 1 μg/mL) challenging. Thus, perhaps cefotaxime behaves differently. Indeed, our results showed low level of emergence of AmpC-overexpressing strains despite 67% of isolates were *E. cloacae, K. aerogenes,* and *C. freundii*. Additionally, cefotaxime use was associated to good results in the clinical cure of infection. But before giving any definite conclusion, further well-designed studies are needed on the topic.

While 3GC could still be a safe option for the treatment of severe wtAE, the increased risk of derepressed AmpC emergence is a potential detrimental effect of these molecules. In a prospective study, including 340 patients harboring an infection caused by an AE or *P. aeruginosa*, authors reported that prior use of cefotaxime, ceftazidime, and piperacillin was associated with 3GCs resistance [36]. In this context, most studies reported an emergence of 3GCs resistance equal or less than 10% [4, 8–11]. However, these studies on AEs focused mainly on bacteremia, without reporting the source of infection. The highest rate of resistance (47%) was observed in ICU patients suffering from pneumonia due to *E. cloacae* [26]. Of note, in our study, we observed only 5.4% of derepressed AmpC, during or after treatment, and all of them were isolated from pulmonary infection. It is noteworthy that cefotaxime was not associated with this selection. However, definitive treatment with cefepime protected from the emergence of mutants. Choi et al*.,* studied 732 patients suffering from AE infection, found no resistance selection under cefepime [8]. So, the emergence of overproducing-AmpC AE in our study is small compared to the number of clinical failures under treatment and was not the main cause of therapeutic failures.

In our study, *E. cloacae* was the most prevalent strain, as in other studies [5, 9, 25, 30]. Overall, studies have not reported differences in outcome between strains, except for the emergence of resistance [8]. In a prospective study, bloodstream infections due to *Serratia* spp. were associated with a significantly higher mortality (29%) when compared to other AE [25]. *E. cloacae* and *K. aerogenes* have long been studied in the same group, but in our study, only *K. aerogenes* was statistically associated with clinical failure. One possible explanation is that clinical failure is more related to the site of infection than to a specific species [8, 12]. In our study *K. aerogenes* were mainly responsible for pneumonia.

Our study has several limitations.

First, infection was defined according to the anatomic site, knowing that it can be difficult to differentiate from colonization in ICU patients. We could have focused our study on bacteremia. But, the infection outcome under treatment depends mainly on its origin. Therefore, we investigated all infection sites deeming that this choice is the most relevant to assess the outcome under treatment. Second, while the patients’ characteristics were similar on admission, the clinical failure group showed a higher severity on the day of AE infection. This can lead to think that the observed results for the cefotaxime are related to bias. However, despite careful selection of the variables included in the multivariate analysis mainly the SOFA score on the day of inclusion, patient severity was not associated with clinical failure, unlike the other variables. In view of this result, we performed an inverse probability weighting analysis, including different factors, such as the SOFA score on the day of AE infection. This analysis confirmed the multivariate analysis results. However, we cannot exclude unmeasured confounding factors, making the results interpretation for cefotaxime very cautious. Only RCTs can answer this question. Third, the secondary endpoint on microbiological failure is difficult to interpret as this study is not randomized and controlled. Thus, mainly the clinical failure group had repeated bacteriological sampling making this point not comparable between the two groups. Fourth, the initial MIC was not measured according to the gold standard. However, according to the CASFM–EUCAST clinical breakpoints, all AE strains included were wild type (MIC ≤ 1 mg/L for 3GCs) [23]. Fifth, no information is available on the antibiotic dosage regimens used or the serum antibiotics concentrations. ICU patients are often underdosed, and physicians counteract this risk by using high-dose antibiotics regimens. Probably, the lack of antibiotic serum monitoring may have influenced the clinical failure rate. Sixth, we did not perform a molecular analysis of the AE isolates to confirm that, for a given patient, the susceptible and resistant strains were related. Indeed, the molecular analyses by Chow et al. suggested that susceptible and resistant isolates were from the same clone in most cases [37]. Finally, testing for AmpC overexpression was only phenotypic but cloxacillin screening for AmpC production is highly sensitive (97.2%) and specific (100%) [38].

## Conclusion

Clinical failure in the treatment of wtAE infection and selection of AmpC derepressed variants is associated with the infection site and the causal microorganism. Additionally, cefotaxime use is probably protective against clinical failure in wtAE infection. Overproducing-AmpC AE are not the main cause of therapeutic failures in ICU patients.

## Supplementary Information

**Additional file 1.** supplementary data on the methods and outcomes of the secondary objectives of the study.

## Data Availability

The datasets used and/or analyzed during the current study are available from the corresponding author on reasonable request.

## Abbreviations

3GCs: Third-generation cephalosporins
ICU: Intensive care unit
VAP: Ventilator-associated pneumonia
WtAE: AmpC-producing Enterobacterales
