# Supplementary material for: Clinical outcome of wild-type AmpC-producing Enterobacterales infection in critically ill patients treated with β-lactams: a prospective multicenter study
Source: Ann Intensive Care. 2022 Nov 17;12:107. doi: 10.1186/s13613-022-01079-5 (PMC9672193; doi:10.1186/s13613-022-01079-5)
Supplement: Supplementary file 1 — Additional file 1. supplementary data on the methods and outcomes of the secondary objectives of the study. [file 13613_2022_1079_MOESM1_ESM.docx]

**SUPPLEMENTARY DATA**

**Supplementary introduction**

AmpC beta-lactamases (AmpC) enzymes are classified as class C in the structural Ambler system. The genes for these enzymes occur naturally in some Gram-negative bacilli as so-called chromosomal AmpC (e.g. in *E. coli*). AmpC expression is constitutively weak and under these conditions it hydrolyzes penicillin G, A and first generation cephalosporins such as cefazolin. Their expression can be inducible (wt-AE, *Pseudomonas aeruginosa*) or not (*Escherichia coli*, *Acinetobacter baumannii*).

AmpC overproduction arises either from a temporary induction of ampC transcription as a response to beta-lactam antibiotics exposure, or from a constitutive dysfunction of the AmpC regulation system due to a mutation in one of the regulatory genes controlling AmpC expression. In case of enzymes overproduction, other beta-lactam antibiotics such as piperacillin, cefuroxime, cefotaxime, ceftriaxone, ceftazidime, and aztreonam can also be hydrolyzed. They also result in resistance to combinations of these antibiotics and substances which are actually intended to inhibit the effect of beta-lactamases. They do not convey resistance to fourth generation cephalosporins.

Beta-lactam antibiotics are AmpC inducer. All beta-lactam antibiotics do not have the same ability to induce AmpC expression. Beta-lactam antibiotics can be classified according to their power of AmpC induction and to their stability regarding AmpC enzymatic activity. However, as soon as the inducer is removed, ampC expression will return to normal. Induction is an in vitro, dynamic, reversible phenomenon with no clinical impact [1, 2].

In contrast to induction, which is a transient phenomenon, selection of resistant mutants producing very high levels of beta-lactamase has been observed in vitro and in vivo [1]. The most common reason for AmpC permanent overproduction in clinical isolates is a constitutive dysfunction of the AmpC regulation system due to a mutation in one of the regulatory genes controlling AmpC expression.

These two different mechanisms are generally misunderstood. Regarding induction, the only risk of therapeutic failure for wt-AE infection would be the combination of a strong inducer with a third-generation cephalosporin [2]. Regarding AmpC permanent overproduction in a susceptible population of wt-AE, there may be mutated bacteria with stable AmpC overproduction, which could be selected by the antimicrobial treatment.

Another mechanism exists linked to AmpC genes localised outside the chromosome on so-called plasmids (plasmidic AmpC). They ensure the constant formation of the enzyme and lie on transmissible gene sections. These can be exchanged between bacteria of the same type or of different types (horizontal gene transfer).

References

1. Goldstein FW (2002) Cephalosporinase induction and cephalosporin resistance: a longstanding misinterpretation. Clin Microbiol Infect 8:823–825. https://doi.org/10.1046/j.1469-0691.2002.00492.x

2. Mizrahi A, Delerue T, Morel H, et al (2020) Infections caused by naturally AmpC-producing Enterobacteriaceae: Can we use third-generation cephalosporins? A narrative review. Int J Antimicrob Agents 55:105834. https://doi.org/10.1016/j.ijantimicag.2019.10.015s

**Supplementary methods**

During the study period, different versions of the CASFM-EUCAST guidelines were used. The 2016 version was used until 6/2/19, the 2018 v.2 version until 4/8/2020, and the 2019 v.2 version until the end of the study.

**Supplementary results**

**Supplementary Figure 1. Flowchart of the study.**


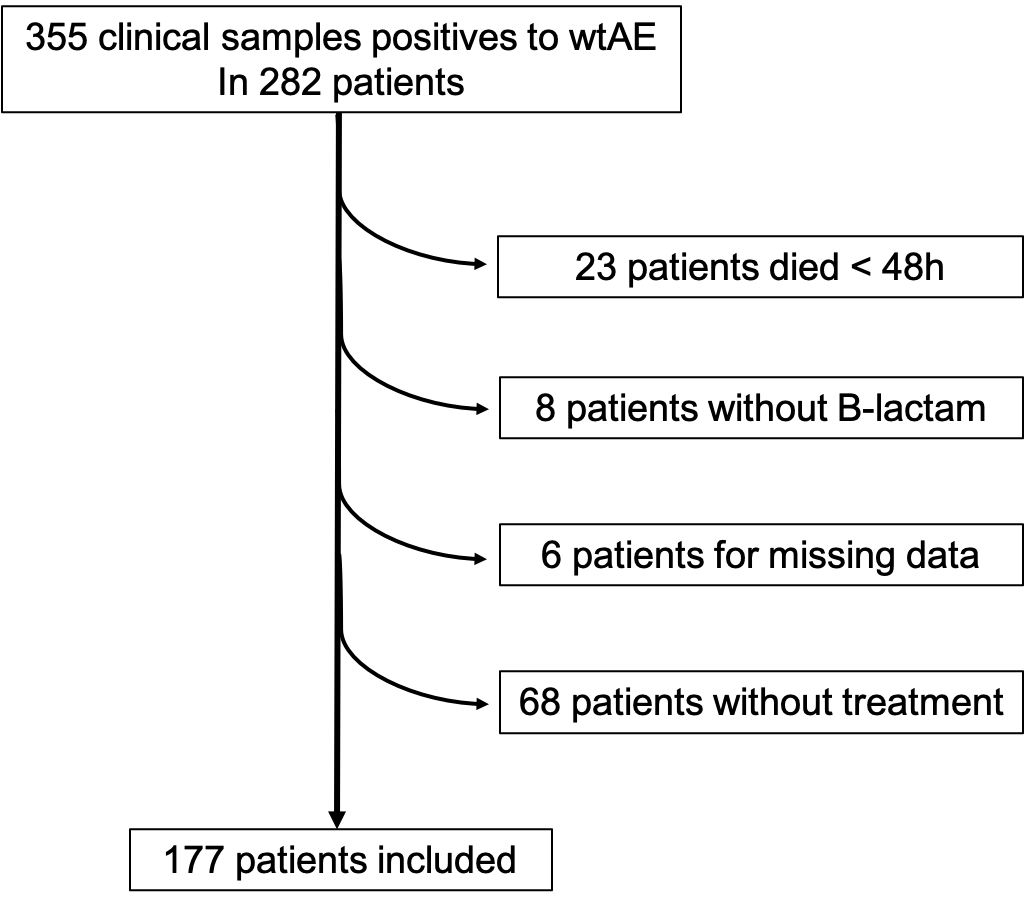


wtAE, wild-type AmpC producing Enterobacteriales.

**Supplementary Table S1. This table shows the characteristics of infections that occurred during intensive care but before wild-type AmpC producing Enterobacteriales infection.**

|  | All population (n=177) | Clinical failure (n=52) | Clinical cure (n=125) | **p** |
| --- | --- | --- | --- | --- |
| Infections before inclusion | 66 (37.3%) | 22 (42.3%) | 44 (35.2%) | 0.373 |
| Number of infections before inclusion | 0 [0 - 1] | 0 [0 - 1] | 0 [0 - 1] | 0.283 |
| Antimicrobial therapy | 0 [0 - 1] | 0 [0 - 1] | 0 [0 - 1] | 0.459 |
| Penicillins | 20 (11.3%) | 9 (17.3%) | 11 (8.8%) | 0.103 |
| Amoxicillin-clavulanate | 20 (11.3%) | 6 (11.5%) | 14 (11.2%) | 0.948 |
| Piperacillin-tazobactam | 11 (6.2%) | 5 (9.6%) | 6 (4.8%) | 0.227 |
| 1 and 2GCs | 1 (0.6%) | 0 (0%) | 1 (0.8%) | 0.518 |
| 3GCs | 19 (10.7%) | 7 (13.5%) | 12 (9.6%) | 0.450 |
| Cefepime | 7 (4%) | 2 (3.8%) | 5 (4%) | 0.962 |
| Carbapenems | 7 (4%) | 1 (1.9%) | 6 (4.8%) | 0.371 |
| Fluoroquinolones | 1 (0.6%) | 0 (0%) | 1 (0.8%) | 0.518 |
| Clindamycin / Metronidazole | 7 (4%) | 1 (1.9%) | 6 (4.8%) | 0.371 |
| ATBs active against anaerobic bacteria | 34 (19.2%) | 11 (21.2%) | 23 (18.4) | 0.672 |
| Aminoglycosides | 17 (9.6%) | 5 (9.6%) | 12 (9.6%) | 0.997 |
| ATBs active only against GPB | 7 (4%) | 1 (1.9%) | 6 (4.8%) | 0.371 |
| Others | 6 (3.4%) | 1 (1.9%) | 5 (4%) | 0.487 |

Note. The two groups are those corresponding to the primary endpoint. For the 3CGs, cefotaxime was used essentially. For carbapenems, meropenem was used essentially. ATBs active against anaerobic bacteria include amoxicillin-clavulanate, piperacillin-tazobactam, clindamycin and metronidazole. GPB: Gram positive bacteria; ATBs active against GPB include vancomycin, linezolid and daptomycin.

ATB, antibiotics; CG, generation cephalosporins.

**Supplementary Table S2. This table summarizes all the data from the study, but in two different groups. The AmpC+ group includes all patients with an AmpC-overproducing *Enterobacteriales*, isolated at any time between the onset of wild-type AE-infection and discharge from the ICU. The AmpC- group includes all patients without AmpC-overproducing isolate.**

|  | AmpC + (n=10) | | AmpC – (n=167) | | **p** |
| --- | --- | --- | --- | --- | --- |
|  | **Nb** | **Results** | **Nb** | **Results** |  |
| Gender (male) | 10 | 10 (100%) | 167 | 124 (74.3%) | 0.065 |
| Age (years) | 10 | 67 [55 - 70] | 167 | 63 [50 - 70] | 0.928 |
| **Past medical history** |  |  |  |  |  |
| Hospitalization during the last 12 months | 10 | 5 (50%) | 165 | 67 (40.6%) | 0.558 |
| Hospitalization delay (months) | 5 | 1 [1 - 3] | 69 | 1 [1 - 1] | 0.356 |
| ATBs during the last three months | 10 | 2 (20%) | 156 | 29 (18.6%) | 0.912 |
| Immunosuppression | 10 | 3 (30%) | 167 | 58 (34.7%) | 0.760 |
| Corticosteroids | 3 | 3 (100%) | 58 | 47 (81%) | 0.405 |
| Immunosuppressive therapy | 3 | 0 (0%) | 58 | 10 (17.2%) | 0.432 |
| Hemopathy | 3 | 0 (0%) | 58 | 1 (1.7%) | 0.819 |
| Extravascular device | 10 | 0 (0%) | 167 | 7 (4.2%) | 0.509 |
| Endovascular device | 10 | 2 (20%) | 167 | 38 (22.8%) | 0.840 |
| Diabetes mellitus | 10 | 5 (50%) | 167 | 41 (24.6%) | 0.075 |
| McCabe score | 10 | 2 [0 - 2] | 166 | 1 [0 - 2] | 0.621 |
| Delay from hospitalization to ICU (days) | 10 | 0.5 [0 - 1] | 164 | 0 [0 - 0] | 0.484 |
| **ICU** |  |  |  |  |  |
| Surgery at admission | 10 | 6 (60%) | 166 | 51 (30.7%) | 0.055 |
| Infection at admission | 10 | 3 (30%) | 165 | 66 (40%) | 0.530 |
| SAPS II score | 8 | 31 [24 - 44] | 132 | 38 [27 - 49] | 0.453 |
| SOFA score at admission | 8 | 6 [5 - 9] | 123 | 6 [3 - 8] | 0.509 |
| **ICU management** |  |  |  |  |  |
| MV | 10 | 10 (100%) | 167 | 141 (84.4%) | 0.177 |
| Duration of MV (days) | 10 | 47 [21 - 59] | 139 | 18 [8 - 29] | **0.005** |
| Renal replacement therapy | 10 | 3 (30%) | 167 | 46 (27.5%) | 0.866 |
| Chest drainage | 10 | 0 (0%) | 167 | 20 (12%) | 0.245 |
| ECMO | 10 | 3 (30%) | 167 | 25 (15%) | 0.206 |
| EVD | 10 | 0 (0%) | 167 | 13 (7.8%) | 0.359 |
| Surgical procedure during ICU course | 10 | 6 (60%) | 167 | 76 (45.5%) | 0.372 |
| Infections before inclusion | 10 | 6 (60%) | 167 | 60 (35.9%) | 0.126 |
| Number of infections before inclusion | 10 | 1 [0 - 1] | 167 | 0 [0 - 1] | 0.067 |
| **Antimicrobial therapy** | **10** | **0.5 [0 - 1]** | **167** | **0 [0 - 1]** | **0.514** |
| Penicillins | 10 | 3 (30%) | 167 | 17 (10.2%) | 0.054 |
| Amoxicillin-clavulanate | 10 | 1 (10%) | 167 | 19 (11.4%) | 0.894 |
| Piperacillin-tazobactam | 10 | 2 (20%) | 167 | 9 (5.4%) | 0.063 |
| 1 and 2GC | 10 | 0 (0%) | 167 | 1 (0.6%) | 0.806 |
| 3GC | 10 | 0 (0%) | 167 | 19 (11.4%) | 0.259 |
| Cefepime | 10 | 1 (10%) | 167 | 6 (3.6%) | 0.313 |
| Carbapenems | 10 | 0 (0%) | 167 | 7 (4.2%) | 0.509 |
| Fluoroquinolones | 10 | 0 (0%) | 167 | 1 (0.6%) | 0.806 |
| Clindamycin / Metronidazole | 10 | 0 (0%) | 167 | 7 (4.2%) | 0.509 |
| ATBs active against anaerobic bacteria | 10 | 3 (30%) | 167 | 31 (18.6%) | 0.407 |
| Aminoglycosides | 10 | 0 (0%) | 167 | 17 (10.2%) | 0.289 |
| ATBs active only against GPB | 10 | 0 (0%) | 167 | 6 (3.6%) | 0.542 |
| Others | 10 | 0 (0%) | 167 | 7 (4.2%) | 0.509 |
| Time between previous ICU infection and wtAE infection (days) | 5 | 7 [4 - 11] | 61 | 8 [5 - 14] | 0.402 |
| Time between ICU admission and AE infection (days) | 10 | 5.5 [4 - 12.5] | 166 | 6.5 [2 - 11] | 0.564 |
| SOFA score at day 0 of infection | 8 | 8 [7.5 - 9.25] | 125 | 6 [3 - 9] | 0.116 |
| MV during infection | 10 | 10 (100%) | 167 | 134 (80.2%) | 0.119 |
| Renal replacement therapy during infection | 10 | 2 (20%) | 167 | 36 (21.6%) | 0.907 |
| Urinary catheter during infection | 10 | 10 (100%) | 167 | 161 (96.4%) | 0.542 |
| Catheter during infection | 10 | 9 (90%) | 167 | 123 (73.7%) | 0.249 |
| Number of catheters | 10 | 2 [2 - 2] | 167 | 2 [0 - 2] | 0.239 |
| Catheters at the end of AMB-therapy | 10 | 8 (80%) | 167 | 81 (48.5%) | 0.053 |
| Number of catheters at the end of AMB-therapy | 10 | 2 [1 - 2] | 167 | 0 [0 - 2] | 0.063 |
| Infected collection | 10 | 1 (10%) | 167 | 56 (33.5%) | 0.122 |
| Infected collection drainage | 10 | 1 (10%) | 167 | 55 (32.9%) | 0.130 |
| Location of wtAE infection |  |  |  |  |  |
| Pulmonary | 10 | 10 (100%) | 167 | 122 (73.1%) | 0.057 |
| Ventilator-associated pneumonia | 10 | 10 (100%) | 167 | 113 (67.7%) | 0.031 |
| Soft tissue | 10 | 0 (0%) | 167 | 14 (8.4%) | 0.340 |
| Abdomen | 10 | 0 (0%) | 167 | 11 (6.6%) | 0.402 |
| Primary bacteraemia | 10 | 0 (0%) | 167 | 5 (3%) | 0.579 |
| CSF | 10 | 0 (0%) | 167 | 5 (3%) | 0.579 |
| Others | 10 | 0 (0%) | 167 | 10 (6%) | 0.426 |
| SOFA score at day 1 of infection | 8 | 7 [5 - 9] | 123 | 6 [3 - 8] | 0.509 |
| SOFA score at day 3 of infection | 8 | 8 [4 - 9] | 123 | 5 [2 - 8] | 0.120 |
| SOFA score at day 8 of infection | 7 | 8 [4 - 9] | 115 | 3 [0 - 6] | 0.103 |
| Microbiological samples after completion of AMB-therapy | 10 | 8 (80%) | 167 | 77 (46.1%) | **0.037** |
| Clinical failure | 10 | 7 (70%) | 167 | 45 (26.9%) | **0.004** |
| Microbiological failure | 8 | 6 (75%) | 77 | 21 (27.3%) | **0.006** |
| Overproduced cephalosporinases | 6 | 5 (83.3%) | 20 | 0 (0%) | **0.000** |
| Recurrent AE infection | 10 | 4 (40%) | 167 | 20 (12%) | **0.032** |
| Time to recurrent infection (days) | 3 | 6 [6 - 11] | 17 | 16 [12 - 22] | 0.071 |
| Recurrent infection with cephalosporinases overproduced | 3 | 3 (100%) | 15 | 0 (0%) | **0.001** |
| New AE infection | 10 | 2 (20%) | 167 | 6 (3.6%) | **0.015** |
| Time between the first and the second AE infection (days) | 2 | 36 [30 - 41] | 6 | 30 [25 - 45] | 1.000 |
| Overproduced cephalosporinases | 2 | 2 (100%) | 5 | 0 (0%) | **0.008** |
| **Outcome** |  |  |  |  |  |
| AmpC+ | 10 | 10 (100%) | 167 | 0 (0%) |  |
| Death during ICU stay | 10 | 6 (60%) | 167 | 62 (37.1%) | 0.149 |
| Length of ICU stay (days) | 10 | 45 [31 - 59] | 160 | 22 [13 - 35] | **0.004** |
| Death during hospitalization | 10 | 7 (70%) | 167 | 66 (39.5%) | 0.057 |
| Time between hospitalization and death (days) | 7 | 54 [40 - 67] | 65 | 26 [16 - 43] | **0.026** |
| Time between wtAE infection and discharge from ICU (days) | 10 | 33 [24 - 42] | 158 | 15 [8 - 24] | **0.003** |
| Death within the first five days of antimicrobial therapy | 10 | 0 (0%) | 166 | 7 (4.2%) | 0.508 |
| Withdrawal life support | 6 | 4 (66.7%) | 62 | 36 (58.1%) | 0.683 |
| Monomicrobial infection | 10 | 6 (60%) | 167 | 65 (38.9%) | 0.187 |
| *E. cloacae* | 10 | 5 (50%) | 167 | 70 (41.9%) | 0.615 |
| *K. aerogenes* | 10 | 3 (30%) | 167 | 29 (17.4%) | 0.313 |
| *S. marcescens* | 10 | 2 (20%) | 167 | 41 (24.6%) | 0.744 |
| *C. freundii* | 10 | 0 (0%) | 167 | 11 (6.6%) | 0.402 |
| *M. morganii* | 10 | 0 (0%) | 167 | 16 (9.6%) | 0.305 |
| *H. alvei* | 10 | 0 (0%) | 167 | 14 (8.4%) | 0.340 |
| Bacterial inoculum | 10 | 26,000 [1,000 – 325,000] | 103 | 10,000 [1,000–1,000,000] | 0.353 |
| **Empirical therapy** | **10** | **10 (100%)** | **167** | **167 (100%)** |  |
| Combination therapy | 10 | 5 (50%) | 167 | 37 (22.2%) | **0.042** |
| Strains susceptible to the empirical AMB-therapy | 10 | 10 (100%) | 167 | 156 (93.4%) | 0.402 |
| Strains susceptible to the β-lactam included in empirical therapy | 10 | 9 (90%) | 167 | 154 (92.2%) | 0.801 |
| Duration of empirical antimicrobial therapy (days) | 5 | 2 [1 - 2] | 98 | 2 [2 - 3] | **0.046** |
| Cefotaxime | 10 | 0 (0%) | 167 | 31 (18,6%) | 0.213 |
| Piperacillin-tazobactam | 10 | 5 (50%) | 167 | 50 (29.9%) | 0.183 |
| Cefepime | 10 | 1 (10%) | 167 | 60 (35.9%) | 0.168 |
| Imipenem | 10 | 1 (10%) | 167 | 7 (4.2%) | 0.390 |
| Meropenem | 10 | 1 (10%) | 167 | 5 (3%) | 0.234 |
| Carbapenems | 10 | 2 (20%) | 167 | 12 (7.2%) | 0.145 |
| Amikacin | 10 | 5 (50%) | 167 | 37 (22.2%) | 0.044 |
| **Definitive AMB-therapy** |  |  |  |  |  |
| Duration of AMB-therapy (days) | 10 | 7.5 [6.25 - 13.5] | 159 | 7 [6 - 10] | 0.708 |
| Piperacillin | 10 | 2 (20%) | 167 | 16 (9.6%) | 0.290 |
| Cefotaxime | 10 | 2 (20%) | 167 | 47 (28.1%) | 0.576 |
| Piperacillin-tazobactam | 10 | 3 (30%) | 167 | 18 (10.8%) | 0.068 |
| Cefepime | 10 | 1 (10%) | 167 | 75 (44.9%) | **0.030** |
| Imipenem | 10 | 1 (10%) | 167 | 2 (1.2%) | 0.161 |
| Meropenem | 10 | 1 (10%) | 167 | 9 (5.4%) | 0.540 |
| Carbapenems | 10 | 2 (20%) | 167 | 11 (6.6%) | 0.114 |

Notes. AMBs active against anaerobic bacteria include amoxicillin-clavulanate, piperacillin-tazobactam, clindamycin and metronidazole. AMBs active against Gram positive bacteria include vancomycin, linezolid and daptomycin.

ATBs, antibiotics; AMBs, antimicrobials; CG, generation cephalosporins; ECMO, Extracorporeal Membrane Oxygenation; EVD, External ventricular Drain; ICU, Intensive Care Unit; MV, mechanical ventilation (invasive); Nb, number of available values for the data; SAPS II, Simplified Acute Physiology Score II; SOFA, Sequential Organ Failure Assessment; wtAE, wild-type AmpC producing Enterobacteriales.
